# Gene Signatures Predict Interferon Response for MS Patients

**DOI:** 10.1371/journal.pbio.0030018

**Published:** 2004-12-28

Multiple sclerosis (MS) can be an unpredictable disease. It develops when the body's immune system attacks healthy nerve cells and disrupts normal nerve signaling. Patients experience a wide range of symptoms—including tingling, paralysis, pain, fatigue, and blurred vision—that can appear independently or in combination, sporadically or persistently. Although symptoms appear in no particular order, flare-ups are common in the majority of patients.

MS flare-ups are commonly treated with beta-interferon. Adverse effects are not uncommon, and, more importantly, a sizable proportion of patients show a reduced response, or no response at all. Given the variability of the disease and treatment response, being able to predict how a particular patient is likely to respond to interferon would help doctors decide how close to monitor the patient or even whether to consider alternative treatments. In a new study, Sergio Baranzini et al. describe a computational model that can predict a patient's therapeutic response to interferon based on their gene expression profiles.

Immune cells typically secrete interferons to fend off viruses and other pathogens. Interferons stem viral infection by inhibiting cell division in neighboring cells—thus preventing the virus from reproducing—and triggering pathways that kill the infected cells. It's thought that interferon therapy may relieve symptoms associated with MS by correcting imbalances in the immune system that lead to disease. Interferon therapy produces changes in the gene expression profile of targeted cells—that is, it inhibits or activates certain genes—which in turn alters the cells' activity.

Blood samples were taken from 52 patients with relapsing-remitting MS (marked by acute flare-ups followed by partial or full recovery), and their RNA was isolated from a class of immune cells called peripheral blood mononuclear cells. After patients started interferon therapy, blood was taken at specific time points over the course of two years. Baranzini et al. measured the expression level of 70 genes—including a number involved in interferon interactions and immune regulation—at each time point.[Fig pbio-0030018-g001]

**Figure pbio-0030018-g001:**
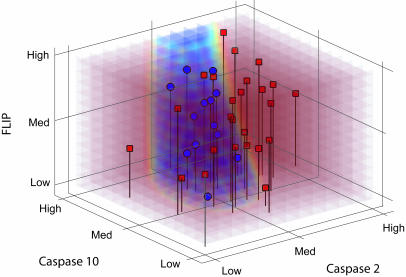
Expression levels of three genes in beta-interferon responders (red) and non-responders (blue)

The authors used statistical analyses to search for gene expression profiles that were associated with patients' therapeutic outcomes. They looked for patterns in analyses of single genes, gene pairs, and gene triplets, and found their model's predictive accuracy increased with gene number. They also looked for genes that showed different expression patterns over the two years based on patient response, time passed, and patient response over time. These analyses identified genes that increased activity independently of clinical response (interferon can activate genes that have no effect on disease), as well as genes that were associated with a good or poor response. Some of these genes were also the best predictors of patient response before therapy was started.

This approach can predict the probability of a good or poor clinical response with up to 86% accuracy. Baranzini et al. offer hypotheses to explain how the observed gene activity might produce the differential responses to therapy—for example, a poor response may stem from downstream signaling events rather than from problems with drug metabolism. But the authors caution that the mechanisms connecting these genetic signatures to specific outcomes—and the mechanisms that produce a positive interferon response—have yet to be established. For now, these patterns should be thought of as markers. Still, these results suggest that doctors could one day tailor MS patients' treatments to their molecular profile, and perhaps take some of the uncertainty out of this capricious disease.

